# Otology

**Published:** 1877-04

**Authors:** 


### IV. Otology.

*A New Method of Treating the Acute Inflammation of the Middle Ear.* Sam. Sexton. (*N. Y. Med. Record*, 1877, p. 17.)

Last September, the writer had under his care a young man who was quite deaf with catarrhal inflammation of the middle ear, and who, at his first visit, stated that, one week ago, while bathing and immersing his head under water, he felt a sharp crack in his ear, and immediately he could hear quite well, but after the lapse of two days the deafness gradually returned. As this temporary improvement of hearing had to be attributed to the pressure of the water upon the membrana tympani, the Doctor decided to try the effect of gentle pressure upon that membrane by means of condensed air. For this purpose he devised a simple instrument, consisting of a soft rubber bulb, connected by a flexible tube to a hard rubber nozzle, which is somewhat olive-shaped and adapted to fit into the entrance of the external auditory meatus. When used, the nozzle is held to the ear by the left hand, and pressed against the opening with sufficient force to make the fitting as nearly air-tight as possible; while the bulb is held in the right hand, to be manipulated as desired. When using the instrument, it is of course never to be forgotten that the membrana tympani is ordinarily never stimulated to action by a greater force than the wave pulses of the air, and that the organ of hearing may be injured by too great force. When thus carefully used, the danger of injury is not so great as by Politzer's inflation, especially in cases where the tubes are freely open. When the instrument is to be used for suction—rarefaction—for instance, to draw out a membrane much depressed, the bulb is simply collapsed by pressure of the right hand before the nozzle is applied to the ear. Upon removing the pressure of the hand, the bulb, by its own elasticity, gradually resumes its expanded condition—thus rarefying the air in the m. externus. If it is desired to give the m. t. and ossicula motion, the bulb is alternately compressed and expanded while the nozzle is in contact with the ear.

The remark is here suggested that if the object of forcing air into the cavity of the tympanum, by means of the catheter or Politzer's experiment, is to remove secretions from that cavity, the result may be frequently of quite an opposite character, *i. e.*, the mucus of the Eustachian tubes may be driven into the tympanic cavity.

If the contents of the tympanum be of such consistence as to be readily driven out at all, it may in many instances be accomplished, to some extent, by pressure upon the m. t. from without.

In infancy and childhood, the difficulties in the way of using the catheter, or Politzer's experiment, are only too well known. But the cavity of the t. may be freed in some measure by pressure from without. My experience is that it always relieves the pain which results from inflammation and the accumulation of serum in the t., where the most free nervous distribution is to be found.

Adhesions can in no other way be so well prevented, or broken up when formed; for the movement of the m. t. may be kept up when other means cannot be resorted to. The extent to which this movement can be carried with benefit is considerable; and when we observe a membrana tympani so concave that it touches the promontory of the t. in one case, or is bulged out by secretions in another, we can judge somewhat of the capacity of these parts for tension without injury. Sometimes the ear is too painful or tender to permit the application of the instrument; its use then is not to be thought of; but the inflamed membrana can be acted upon in most cases without pain or injury.

It is not unlikely that the frequent (daily) pressure upon the m. t. in many cases may excite the movement of serum along the e. t. towards the pharynx, and thus keep the t. free of hurtful collections. That this is the natural outlet for the secretions of the t. cavity, there can be no doubt. When tinnitus aurium depends upon the adhesions between the malleus and m. t., and between the ossicula and the stapes and fenestra ovalis, may not much benefit arise from exercising, as it were, this mechanism? The air forced into the t. cavity through the Eustachian tubes does not perform this so well, and cannot be done with the same ease and gentleness.

The frequency of the applications must be determined by the results as derived from experience.

The congestion of the m. t. is not as marked after this operation as is sometimes the case after syringing—usually, there is none. The hearing power in nearly all cases of deafness will be increased in as great a degree as by any other means we are acquainted with, and in many instances benefit is found where other means do not avail. I am aware that many otologists have made valuable suggestions as to the use of condensed and rarefied air, but I believe none have recommended its use, in acute aural catarrh, in the manner here indicated.

*Nitrate of Amyl in Tinnitus Aurium.* MICHAEL. (*Arch. Ophthalmol. and Otol.*, vol. v., 4.)

In many diseases of the ear, the tinnitus is the symptom which causes the patient more annoyance than the impairment of the hearing; and in a large number of cases it does not subside with the disappearance of the causative disease. Dr. Michael, of Hamburg, has tried the nitrate of amyl for the relief of the tinnitus. He was led to employ this remedy on account of its well-known sedative action upon the sympathetic system, especially the vaso-motor nerves, and the fact that many forms of tinnitus are caused by an irritative state of the auditory nerve, or are the result of hyperæmia, or anæmia of the brain, or the internal ear.

He administered the nitrate of amyl in twenty-seven cases. The results were as follows: A greater or less degree of improvement was obtained in nineteen cases. Among these were three in which the tinnitus disappeared entirely from one ear and was diminished in the other ear. In four cases, a by no means inconsiderable improvement of the hearing was observable. Three cases passed from observation. In the majority, there was an otitis media hypertrophica; of the cases not improved, one; and of those improved, two were diseases of the labyrinth.

From one to five drops were inhaled at a sitting. The inhalation was continued during the appearance of the usual symptoms (flushing of the face, injection of the blood vessels of the eye), and suspended on the occurrence of vertigo. In all the cases improved, the tinnitus was increased during the period of inhalation. As the flushing of the face disappeared, the tinnitus diminished and became less than before the administration. In some cases the improvement lasted but one hour; in others, several weeks, but usually from two to ten days. The effect of a second inhalation lasted longer than that of the first application, provided it did not follow the latter too soon; an interval of two days at least must be allowed. The remedy, of course, is not appropriate for acute catarrhal cases, or others in which the tinnitus is evidently of a mechanical origin.

*A New Artificial Membrana Tympani.* L. TURNBULL. (*Phil. Med. and Surg. Rep.*, Dec., 1876.)

The modifications suggested by T. consist in making the stem of steel, and covering it with a thin layer of rubber; at the end of this stem is a light metal ring, bearing stretched across it a thin rubber membrane. The advantage claimed for this artificial drum is, that the stem being attached, not to the centre, but to the circumference of the membrane, the latter can respond better to the impulses of the waves of sound and convey their whole intensity to the inner ear.

*On the Diagnosis of the Anomalies of the Conduction of Sounds in the Ear.* PROF. GRUBER. (*Allg. Wiener Med. Ztg.*, 1877, No. 7.)

The conduction of the sounds may, among other causes, be disturbed by an irregular tension of the membrana tympani. An abnormal relaxation, or an excessive tension of this membrane must interfere with the normal function of the organ of hearing. The relaxation of the membr. tymp. (or of a portion thereof), can be diagnosed by inspection. If the patient forces air into his ear by the Valsalvian experiment, while we are looking at the illuminated drum-head, we can plainly see the membrane make a decided outward movement. In several such cases, it was also ascertained that the hearing was decidedly improved after the experiment (*i. e.*, after the membr. tymp. had obtained a better tension), but that this improvement gradually disappeared again when the membrane returned to the former relaxed state.

The opposite condition of the membrana tympani, its increased tension, in many cases, cannot be ascertained by ocular inspection, because we have no efficient means of relaxing an abnormally tense drum-head. In this case, the tuning fork proves to be of great value.

A few years ago, Gruber found that the tone of a tuning fork held opposite the external meatus, grew fainter when he inflated his middle ear by the Valsalvian experiment, and thereby increased the tension of the membrana tympani; and as soon as the inflation was discontinued and the membrane returned to its natural condition, the tone of the fork became louder again. Consequently, if to a patient the sound of the fork held opposite his ear does not decrease in intensity during, and again increase after, the Valsalvian experiment, we can infer that, the tension of his membrana tympani has not been increased by the inflation of air, because it has before already been abnormally great. This experiment, however, is not free of errors, because we are wholly dependent upon the patient's statements, and his capacity of making correct observations. These statements must, therefore, be controlled by another test. For this purpose, the tuning fork is placed on the middle of the forehead, and again the patient makes the Valsalvian experiment.

Where no abnormal tension existed in the membr. tymp., the patient will hear the sound of the tuning fork better during the experiment, because the waves of the sound that have been conveyed through the bones of the skull to the air of the middle ear are reflected back on the membr. tympani, while its tension has temporarily been increased by the inflation. As soon as this artificial tension is reduced by the cessation of inflation, the waves of sound which have reached the middle ear are no longer reflected, but pass out through the normal membr. tymp. to the air of the external meatus; the patient then hears the sound considerably fainter. In case of an exeessive tension of the membrane the intensity of the sound of the tuning fork put on the head will not be materially altered by the inflation of the middle ear, because it does not materially change the tension of the membr. tympani. Thus, by this double test with the same instrument (tuning fork), and during the same experiment (Valsalva's inflation) the patient receives, apparently, conflicting impressions, according to whether the tuning fork is held opposite his ear or put on his head.

